# GERIATRIC MEASURES AS PREDICTORS OF 1-YEAR MORTALITY IN MAJOR SURGERY PATIENTS

**DOI:** 10.1093/geroni/igz038.1670

**Published:** 2019-11-08

**Authors:** Victoria L Tang, Kenneth Covinsky, Emily Finlayson, Bocheng Jing, John Boscardin, Sarah Ngo

**Affiliations:** University of California San Francisco, San Francisco, California, United States

## Abstract

A growing proportion of older adults are undergoing major surgery despite the higher risk of post-operative mortality. Geriatric measures (i.e. physical, cognitive, and psychosocial function) are often not included in studies evaluating post-operative outcomes in older adults. Our goal was to determine the association of geriatric measures and 1-year mortality in older adults after major surgery. We analyzed longitudinal data from the Health and Retirement Study linked to Medicare claims (N=1364 participants), age ≥ 65 and who underwent abdominal aortic aneurysm [AAA] repair, coronary artery bypass graft [CABG], or colectomy. Our outcome was mortality within 1 year of the major operation. Predictors included the following geriatric measures: dependence in activities of daily living (ADL), dependence in independent activities of daily living (IADL), mobility ability, and dementia, and depression. We analyzed using multivariate cox proportional hazard models. Mean participant age was 76±6 years, 56% were women, 11% underwent a AAA repair, 50% CABG, 40% colectomy; 18% died within 1 year of their major operation. After adjusting for age, comorbidity burden, surgical type, gender, race, wealth, income, and education, the following measures were significantly associated with 1-year mortality: depression (adjusted HR (aHR): 1.53, p=0.03), dementia (aHR: 1.90, p=0.03), >1 ADL dependence (aHR: 2.35, p<0.01), >1 IADL dependence (aHR: 1.95, p<0.01), and inability to walk several blocks (aHR: 1.69, p<0.01). In this cohort, 18% of participants who underwent major surgery died within 1 year and function, cognition, and psychological well-being were significantly associated with mortality. These measures should be incorporated into pre-operative assessment.

